# Hospital and Institutional News

**Published:** 1913-05-31

**Authors:** 


### HOSPITAL AND INSTITUTIONAL NEWS.

### MR. McKENNA AT CARDIFF MENTAL HOSPITAL.

Last week the Home Secretary, Mr. McKenna, paid a visit of inspection to Cardiff Mental Hospital, where an hour was spent in the institution. Dr. Edwin Goodall, the medical superintendent, then conducted the Home Secretary over the new pathological research laboratory, and summarised the exhaustive study during a generation of the microscopic changes of the brain of the mental patient. He pointed out the need for the chemist and physiologist, as well as for the pathologist and bacteriologist, and consequently for a research institute. Mr. McKenna was informed of the immediate need for a research worker in physiology, and for a library of books and technical periodicals, which might form a department under a director, who should be a whole-time expert in experimental medicine. This implies a scheme for all mental hospitals combined with a complete school of medicine, which might inspire State aid for the expense of carrying out such research work in the pathology of insanity. Dr. H. A. Schölberg is the present pathologist, and Dr. Stanford the research chemist, who conducted Mr. McKenna over their departments. It is not only the visit itself, but the results which may flow from it, that make last week's function possibly a historic date in the evolution of the mental hospital, by opening out new possibilities of organised and specialised mental research.

### LORD ROWALLAN'S SUGGESTION.

We publish this week a remarkable letter from Lord Rowallan, chairman of the Victoria Infirmary, Glasgow, urging the importance of securing for hospital patients some of that mass of unsold daily and weekly newspapers which is now consigned to the waste-paper dealer. The suggestion itself is not new, but Lord Rowallan has begun to set the ball rolling by approaching, he tells us, certain newspapers and hospital authorities for putting the idea into practical working in London as a start. Naturally, the newspapers are no less pleased at the chance of securing more readers, than are the hospitals at having more to read, and we hope that Lord Rowallan's suggestion will be assisted by the prompt calling of a meeting of representatives of both parties to discuss the best and quickest way of diverting unsold copies to hospital wards. While welcoming criticisms and suggestions from all our readers, we suggest that Lord Rowallan should call a meeting himself next week, and, to facilitate his task, London hospitals and editors wishing to co-operate should write to us or direct to him without delay.

### CENTENARY OF THE LONDON ORPHAN ASYLUM.

On May 29 the centenary dinner of the London Orphan Asylum, Watford, took place, with Prince Arthur of Connaught in the chair. A statement of the work achieved in the past hundred years was made by Mr. A. P. Blathwayt, the chairman, and Mr. E. H. Bousfield, the treasurer—a statement of which any institution would feel proud. The shelter afforded by the Asylum lasts, on an average, for six years, and every corner of the Empire has provided candidates for its protection, from the ranks of professional men, merchants, master tradesmen, and clerks. Its aim, in short, is to house, feed, clothe, educate, and to provide an outfit for orphan children at a cost—since subscriptions provide a limit of the work that may be attempted—of £15,000 a year, which is raised from voluntary subscriptions, as there is no endowment fund. When we remember that the practical chances of any citizen in the United Kingdom are about forty to one against his being able to provide these essentials for himself, the need of the fatherless has changed from a matter of sentimental to one of national importance. To-day is the public's chance of giving a new lease of life to this invaluable work.

### INSURANCE FEES GARNISHEED: IMPORTANT DECISION.

Judge Parry on Monday gave his judgment at the Lambeth County Court as to whether the fees paid to a doctor under the Insurance Act could be garnisheed. The case was reported in the last issue of The Hospital, p. 243. Judge Parry was of opinion that the fees could be garnisheed, but the further point raised by the plaintiff's solicitor that he was bound to order the payment out of Court of the whole amount, £33, had been raised in cases in the High Court, when it was shown that the County Court Judge had full discretion. One of his duties was to temper "the wind to the shorn lamb." He, therefore, decided to use his discretionary powers in giving judgment. Dr. Michael, the defendant, said he had neither any private means or practice, and was dependent on the patients on his panel for his livelihood. Judge Parry thereupon ordered that plaintiff should receive £10 and costs out of the sum deposited in Court, the remainder to be paid to Dr. Michael.

### A COMEDY BUT YET A SCANDAL.

Politicians have often been known to throw grave doubt upon the methods of the scientific world. We may, therefore, venture to call the attention of men of science to a cartoon by Sir F. Carruthers Gould which appeared in the *Westminster Gazette* of the 27th instant entitled "Lloyd George and the White Dragon." In this Mr. Lloyd George is represented as a warrior armed with a sword labelled "Sanatoria" and a shield inscribed "Insurance Act" mounting a flight of steps, on the top of which is a dragon with a human skull for a head and "Consumption" written upon its rump. What members of the profession are responsible for the burlesque of fact all this represents? Underneath the cartoon a quotation is given from a speech by Mr. Lloyd George at Criccieth on the 25th instant. "He remembered what had happened in his own home, and looking to the east, west, north, and south, he saw everywhere dwellings, some humble, some ample, where this disease had found a tranquil home and had left it desolated. As he thought of these village tragedies the beauty of the landscape faded, the verdure withered, and he could see the grim monster still stalking defiantly through the glen challenging any power to save from his poisonous fangs the victims he had marked for destruction. They were there that night to accept that challenge." Not only Mr. Lloyd George, but Sir F. C. Gould and the *Westminster Gazette* must lose caste in the sense of seriousness and responsibility by the burlesque of fact thus served up as a political cartoon for distribution, we presume, throughout the length and breadth of the land. The point, of course, is that sanatoria are merely a palliative for tuberculosis. That is why it is so essential that great caution should be exercised before the Treasury pass large sums of money to be sunk in the erection of sanatoria. The *prevention* of tuberculosis, as opposed to its relief when established, is no whit advanced by Insurance sanatoria, and not much by any other provision of the Insurance Act.

### MR. LLOYD GEORGE DISCREDITS HIMSELF.

Heaven knows that the tragedy of tuberculosis in the family or the individual is grievous enough. The prolonged suffering, the brave continuous fight against the disease on the part of the patient, the almost uninterrupted cheerfulness with which the fight is sustained, the joy of recovery in the very few cases where this result has been attained in the past, or the sudden death at the very moment when the patient felt most certain that a cure was within his grasp, are known to us all. But we must protest against the position taken up by Mr. Lloyd George in his speech and by Sir F. C. Gould of claiming that Mr. Lloyd George is a modern saint, who, by moving Parliament to vote millions for the erection of sanatoria, has at length slain, to quote Mr. Lloyd George's words, "the grim monster still stalking defiantly through the glen, challenging any power to save from his poisonous fangs the victims he had marked for destruction." Such a claim is unreal and indefensible in the mouth of a public man, which must discredit him as a serious politician, and is to be condemned because it may lead the victims of tuberculosis to believe that the sanatorium is a panacea for all their woes.

### A HISTORY OF NURSING.

Our review of this book, which appeared in The Hospital of March 8 last (p. 622), has called forth a letter from Miss Adelaide Nutting, of Teachers College, Columbia University, which will be found in our correspondence columns. Miss Nutting, whom we have the pleasure of knowing personally, is one of the ablest and most knowledgeable of women workers among the sick. We have the greatest respect and admiration for the work she has done and also for the work which has been done in the poorer districts of New York, especially by Miss Dock. In such circumstances our regret is, as hospital managers and British nurses throughout the country will realise, that two ladies of such great qualities should have been so entirely misdirected and prejudiced by the small clique who are responsible for so many things which have proved most regrettable in British nursing affairs. We hope Miss Nutting's letter and our reviewer's comments thereon will be read, as they will certainly be appreciated, by those who know most about nursing matters throughout the British Empire.

### INFIRMARY SCRUBBERS AND SUPERANNUATION.

Judge Parry, at Lambeth County Court, has heard an interesting case in which Mrs. Alice Richards, for six years in the employment of the Southwark Board of Guardians at their infirmary at Dulwich as a scrubber, claimed the return of her superannuation contributions, amounting to £4 2s. 11d. For over five years Mrs. Richards, then an unmarried woman, was never late, and only absent from duty on one day, but from her marriage in August to her dismissal in October she was either late or absent from duty five times. She was not alone in her abstentions, and consequently the Guardians were compelled, in order to maintain discipline and to ensure the smooth working of the institution, to put into force the rule that abstention from duty would cause dismissal. On October 19 the Matron had to warn seven scrubbers, amongst them being Mrs. Richards, but the very next day she failed to attend. The Guardians held that this was wilful misconduct, and that under these circumstances the contributions should not be returned. They further pleaded that, as Mrs. Richards would not be entitled to a return of her contributions if she resigned, she had wilfully stayed away in order to be dismissed. The Judge's sympathies were with Mrs. Richards, for whom he gave judgment for the full amount, with costs. He thought the Guardians could have so arranged matters that plaintiff would not have to stoop to a subterfuge in order to get a return of this very small sum. He was glad the Guardians had not made out their case. So are we.

### SHOULD KITCHENS BE ON THE TOP STOREY?

It is noteworthy that at the new King's College Hospital buildings, which are the latest, if not the last word, in hospital construction, the kitchen is, as heretofore, on the ground floor. Yet, the advantages of a kitchen at the top of an institution are many, including the absence of cookery smells throughout the building and the much better light obtained, with the consequent saving of gas and electric current. This matter is not merely one of theory, for it has been put to a practical test, as Mr. J. H. Johnson, the secretary of the Royal Westminster Ophthalmic Hospital, can testify. The kitchen which was built at the top of his institution some time ago has now been in use for several months, and he expresses great satisfaction with it. For the conveyance of heavy supplies, such as sacks of potatoes, a hand-hoist is fitted. For all other articles a small electric-service lift is quite sufficient. So far as the distribution of meals is concerned, it matters little whether the base of operations be at the top or at the bottom. A further advantage of the high kitchen is that the quarters of the kitchen staff may be placed adjacent to it, thus forming a distinct unit of this department, and saving the time and trouble of that weary drag upstairs which is the usual lot of the kitchen-maid. Possibly, in a very large hospital, having a constant influx of stores, the fact that these had all to be carried to the top might cause some inconvenience, especially in view of the fallibility of lifts. But in a small institution this does not apply, and the idea of a light and airy kitchen ventilating to the upper atmosphere is very attractive. Why these considerations were outweighed by others Mr. W. A. Pite, F.R.I.B.A., might usefully explain, for there is no course so good as not to have many serious objections.

### PASSING TRAINS AS A FORM OF VENTILATION.

A further interesting point about the King's College Hospital buildings at Denmark Hill is that the railway runs close to the back of the hospital, and, in one place at least, trains pass at only about a dozen yards from the end of the wards. This is not regarded altogether as a disadvantage by the architect, Mr. W. A. Pite, who considers that each train as it rushes past draws away a considerable quantity of air, thus causing a constant ventilation through the wards. It remains to be seen whether this advantage will be sufficient to counterbalance the inconvenience which, it is feared, will be caused by the noise of the trains (see an article on "The Patients' Point of View," elsewhere), both to the patients trying to sleep and to the doctors going their rounds, especially as the latter will be continually using the stethoscope. We have before heard of a man with phthisical tendencies who purposely chose to live in a house at the back of which trains constantly ran, on the ground that he obtained thereby a constant flow of fresh air. We are not aware, however, that this theory has been the basis of any scientific observations or experiments. Perhaps King's College Hospital will be able to supply these at some future date.

### THE LIMIT OF 2,000 PATIENTS.

We are delighted to observe that Dr. P. Rockliff moved at a meeting of the London Insurance Committee last week that it is undesirable that any panel doctor should undertake to treat more than 2,000 insured persons, and that the panel doctors should be so informed. We are still more delighted to have the Medical Benefit Sub-Committee reporting that they view with great doubt the possibility of adequate treatment being provided in cases where an exceptionally large number of insured persons has been accepted by each doctor; for if these things are said in a sub-committee, what may not be done at last in the committee itself? In its admirable report the Medical Benefit Sub-Committee point out two things. First, that by the regulations if the continuance of a doctor on the panel is reported as prejudicial to the efficiency of the medical service the Insurance Commissioners may hold an inquiry, which might be interpretated to mean that a panel doctor with more than 2,000 patients was prejudicial, as he certainly is. Second, that were the insured persons in London distributed equally between the panel doctors, each doctor's list would average 1,100 in point of patients, and some £350 in point of income, apart from any fees derived from dependants and non-insured generally. The sub-committee have put their view into practice by recommending that the surplus, £34,000, which is over in respect of the 400,000 unaccepted insured persons in London, be distributed proportionately only among those panel doctors who have less than 2,000 insured persons on their list. But on the question of its legality the proposal has been withdrawn.

### VISITING PATIENTS WITHOUT THEIR CONSENT.

A woman in domestic service has been awarded at Lambeth County Court £25 against a medical man, Dr. J. A. Tolmie, for damages for assault. In giving evidence the woman stated that while in domestic service she fell downstairs and injured herself. The insurance company in which her employer was a policy-holder sent the doctor to examine her. She received no notice of his visit, and was asleep in bed when he came. He roughly pushed passed her mistress, who opened the door, awoke and examined her, and, the witness added, tore off a plaster and strap which her own doctor had applied, thereby causing her much pain. The doctor said the woman's mistress invited him to the house, and that he removed the plaster at the plaintiff's request. He had made no appointment to visit her; that was a common thing; 75 per cent. of such cases were done under the same terms. In reply to the Judge's question, "You suggest that doctors visit patients without first obtaining their consent?" Dr. Tolmie replied: "It is done in these cases." To this the Judge retorted that the defendant had set up a new idea as to the rights of doctors, which, from his knowledge of the profession, he thought was a libel on doctors. But Judge Parry, we believe, is not likely to come under the provisions of the Workmen's Compensation Act. It is when doctors visit patients not primarily to serve them, but as the agents of insurance companies, naturally anxious that the alleged injury shall not be proved too severe, that doctors may be induced to visit without the consent of the patient. It is the consent of the insurance company that inspires them, and may lead to want of tact, or even worse.

### MENTAL HOSPITALS AND THE VALUE OF PROPERTY.

What on earth is the reason which makes every proposal for building a new institution in rural areas to be greeted with a chorus of protest from the local property owners, on the ground that it will tend to decrease the value of local property? The latest example of this curious fear is seen in South Buckinghamshire, where strong protests have been made at the attempt of the Middlesex Mental Hospital Committee to secure a site for a mental hospital. A moment's consideration shows that a mental hospital is an institution which requires a site large enough to possess all the amenities of a park, and, as late designs have shown, a type of building that may generally compare on equal terms with the average modern country house of any importance. Æsthetically, then, a new mental hospital may be said to add a park and a palace to the neighbourhood. The park, like the palace, moreover, is not only ornamental, but preserves the patients, who, in seven cases out of ten, are as far removed from the popular idea of "a lunatic" as possible, from obtruding their presence on their timid fellow-mortals outside. The park is at once an ornament and a barrier. On the economic side such a new institution, whether sanatorium, mental hospital, or what not, invariably brings much trade to the neighbourhood, and where the example of the living-out system, as practised at Leavesden, is followed, as it increasingly is to-day, the value of land is increased by the steady demand for cottages beyond the limits of the hospital estate, not merely for the staff who live out, but for families or the relatives, who so often come to live with them. In the face of these undoubted facts we very much hope that the Buckinghamshire protesters will let us know on what grounds their fears that property will be reduced in value if the proposed institution be built are based? If not, judgment by default must go against them.

### A DELAYED MENTAL HOSPITAL APPOINTMENT.

A peculiar position has arisen at the North Wales Counties Mental Hospital at Denbigh with regard to the appointment of a medical superintendent to succeed Dr. W. Stanley Hughes, who was recently appointed to a similar post at the Salop Mental Hospital. The committee of visitors met recently to consider applications. There had originally been nine applicants, but these had been reduced to three—Dr. A. E. Evans of Prestatyn, Dr. Isaacs of Colney Hatch, and Dr. Bodvel Roberts of Natsbury Mental Hospital, St. Albans. These three gentlemen were interviewed, but, after a long discussion, the committee decided to postpone the appointment for a month with a view to eliminating the age-limit, thereby giving other applicants a chance to stand for the position.

### DR. WILSON AND THE PENGUINS.

In Commander Evans' lecture at the Albert Hall last week, on Captain Scott's Expedition, he paid a striking tribute to Dr. E. A. Wilson, whom he described as "our Solomon." "The first winter," he added, "seemed to pass very quickly. Everyone was busy at his special subject. Dr. Wilson, the chief of our scientific staff, helped all of us. To "Uncle Bill" we all went for sound practical advice. Wilson was the friend and companion of Captain Scott and, indeed, of all the Expedition." It was Dr. Wilson who formed one of the party to Cape Crozier, the object of which was to observe the incubation of the Emperor Penguins at their rockery. Half-a-dozen eggs were collected, three of which are still possessed by the survivors, and Dr. Wilson was much amused to pick up rounded pieces of ice which the stupid birds had fondly cherished as if they were eggs. It is not only penguins, however, which fondly cherish colourable imitations of the real thing, whether in the sphere of art, medicine, science, or morality, and men, like penguins, are not too much pleased to have their "stupidity" pointed out to them.

### THE ENDOWMENT OF CANCER RESEARCH.

The tide in favour of the endowment of cancer research is still flowing as may be seen by the gift of £10,000 which Mr. Edwin Tate has sent to the Honorary Treasurer of the Imperial Cancer Research Fund. Luckily the impulse generously to endow this particular branch of investigation has not been the sole desire of the public, which also of late has shown much favour to tropical medicine, and, of course, to tuberculosis. It may be admitted that the old public favourites, children's hospitals and hospitals for incurables, may have suffered some diminution in public support of recent years, but the fact is that the population to-day is sufficiently large to supply an almost infinite number of minor publics, each strenuously devoted to some special branch of charity or scientific investigation. This enables an institution which has received an enormous bequest nominally to disappear as appealing for funds from, say, the notice of newspaper readers, while soliciting public support as actively as ever through another advertising medium, of which the various mechanical forms of traction above and below the surface of the earth seem to have been most favoured at present.

### THE EXTENSION OF COVENTRY HOSPITAL.

Two new wards have become ready for occupation at the Coventry and Warwickshire Hospital in connection with the King Edward VII. Memorial Wing, and increase the accommodation by fifty-two beds. The wing consists of a men's ward on the ground floor, a women's ward on the first floor, with the kitchens above, and adjoining them the larders, grocery stores, and servants' dormitory. Another feature of the new wing is an electric lift which serves all floors. In addition a nurses' dining room and a new theatre have been provided, while the alterations to Gulson Ward will add eight beds to this part of the hospital. The expenditure on the new wing and the alterations has amounted to rather more than £9,000 without furnishing. The complete extension scheme has been estimated at a sum of £45,000, of which sum £30,000 has been subscribed.

### A BREWER'S MUNIFICENT BEQUEST.

We are glad to record a very large bequest to the Burton-on-Trent Infirmary. Mr. John Wood, the honorary secretary of the institution, has received a letter from the solicitors of the late Mr.

Robert Ratcliff, director of Bass, Ratcliff and Gretton, stating that the deceased had left £20,000 to the infirmary. It is interesting to add that Mr. Ratcliff never signed the will which he had prepared. It appears, however, that his sons have decided to carry out what they knew to be their father's intentions. This hospital has always received a large share of support from the firm, whose name is now a household word, and it would have been strange indeed, and contrary to all expectation, if Mr. Ratcliff had not made provision for the institution in his will. That that provision was never legally secured proves again how easily promises or intentions may go astray, unless, indeed, relatives prove willing, as in this case, to carry out unfulfilled intentions.

### THE DEARTH OF CONVALESCENT HOMES FOR CHILDREN.

Mr. E. G. Gauntlett, surgeon to the Paddington Green Children's Hospital, must surely have been carelessly reported when the lay press attributed to him the statement that "there are several convalescent homes for adults, but not one for children" from London. Paddington Green Children's Hospital itself, to say nothing of the Victoria Hospital for Children, the East London Hospital for Children, Shadwell, the Queen's Hospital for Children, Hackney Road, to go no further, all possess convalescent homes. On the other hand, it is certainly true that convalescent treatment is not nearly sufficiently extended, while it is needed more than ever now that every effort has been made, and made with success, to reduce the period during which patients are warded. The provincial hospitals are, as a whole, probably even worse organised in this respect, and the various units are left in somewhat a haphazard condition instead of being carefully co-ordinated. The result is, and this applies to London hospitals in their degree also, that much work done in the wards is undone on the discharge of the patient. That, however, is not the same thing as saying that there is no convalescent home for children from London. What did Mr. E. G. Gauntlett, as interpreted by his reporter, really mean? If he meant a large convalescent home to serve all or a selected group of London children's hospitals, the idea is worth discussing at greater length.

### THE SANATORIUM SECRETARY.

An interesting state of affairs in relation to the work of sanatorium officers and of the institution in respect of the Insurance Act was revealed at the recent annual meeting of the Kelling Sanatorium. Mr. R. E. Horsfall alluded to the need for a more extended system of approval than the short three months at present given by the Local Government Board, if the sanatorium was to come to any permanent arrangement with authorities desiring to co-operate with them. So far temporary arrangements with local authorities had been renewed as periodically as the official approval. No permanent rules as regards insured patients' beds have been laid down, but at present applications are restricted to patients in Norfolk or London. A permanent scheme is contemplated which, he hoped, would include some provision for advanced cases. Dr. Burton-Fanning urged the importance of "a sort of Scotland-Yard arrangement, under which suspicious cases were found out," and gave a very high tribute of praise to the secretary, Major Dalton, for his indefatigable work. The public has heard less about the work of a sanatorium secretary than probably of any institutional secretary at all. Yet this is the official who can best popularise the work of the institution and the medical staff upon which successful voluntary support depends. The opportunity is only waiting for some sanatorium secretary to come forward and to supplement the experience given by medical men of the working of the Act by his own version of the way in which the administrative side of sanatorium work is affected by it. The chance of a valuable contribution on these lines is only waiting. Where is the man sufficiently keen to act on it?

### THE SWANSEA CRISIS MORE HOPEFUL.

A special meeting of the board of management of the Swansea Hospital was held on Thursday, last week, to consider three important recommendations of the finance committee for curtailing the hospital's work owing to the financial impasse. "Pending the receipt of subscriptions now withheld by public bodies and employees," says the finance committee's report, which was read by Mr. W. D. Hughes, the secretary, "the committee recommend the reduction of beds in the hospital to a maximum of 100, and that the convalescent home should revert to its former use and be strictly limited to the number of patients which can be maintained on the income derived from its endowment fund; further, that all building operations be suspended in as far as practicable." In response to Mr. Hughes' appeals the organised workers of Swansea, through their labour association, have been advised to pay their subscriptions to the hospital for the current year, as have also all the trade union branches at Briton Ferry. "If," Mr. Hughes added, "the dispute between the medical profession and the workmen were settled in the course of the next few weeks, there was every reason for hoping that the contributions from employees would not only keep up to the present level, but be substantially increased." Mr. R. W. Jones, chairman of the finance committee, in submitting the report, said that, although the committee had recommended the closing of beds, the prospects of the institution for the time being had become brighter owing to the workmen's decision to continue paying their contribution for the current year. It was decided to discuss the recommendations in sections, and Mr. Jones therefore moved first the adoption of the recommendation respecting the curtailment of beds, which Colonel Morgan seconded. Mr. Charles Tuckfield urged that they were not justified in limiting the usefulness of the hospital to the slightest extent, and that a decision might with advantage be deferred. His amendment to this effect was carried. Dr. Edgar Reid, chairman of the honorary medical staff, said they had a meeting the previous evening, and certain information was put before them which led them to believe there was a hopeful movement outside for the solution of the differences.

### HOSPITAL SUNDAY IN LONDON, MAY 25, 1913

The newspapers, possibly owing to the death of the Secretary of the Fund, Sir Edmund Hay Currie, did less than justice this year to this Fund and the eloquence of the preachers on its behalf. The Bishop of London, for instance, preached a sermon at St. Stephen's, Westbourne Park, on Sunday morning which was probably the simplest and yet the most irresistible appeal which was ever uttered from the pulpit on behalf of the hospitals. Not a line of this sermon appears, so far as we have noticed, in any daily paper, a fact which in these days does honour in one sense to the Bishop. The subject of the sermon was love, taken from the First Epistle of St. John, fourth chapter, seventh verse. We shall hope to publish the substance of this most touching and important address, and can only regret that it has escaped the notice of our colleagues in the lay press, who would, we are confident, have found the "copy" of the best.

### NATIONAL INSURANCE AND LONDON HOSPITAL NURSES.

In a letter addressed to the nursing staff and probationers of the London Hospital Mr. Holland shows cause why, in the opinion of the London Hospital authorities, they are able to train a nurse in two years, whereas other nurses say they need three. The reason is that the London possesses exceptional opportunities and a system specially organised to guarantee effective training to each probationer within the period of two years. Mr. Holland insists that the special merit of the two years' system at the London is that the nurses there have work involving responsibility put upon them within two years, and nothing develops a woman or man more than responsibility. Mr. Holland goes on to state that the Insurance Commissioners have agreed to pay the hospital nine shillings for every nurse; that is the amount payable to panel doctors for each insured person on their books. One of the physicians of the London Hospital has agreed to go on the starred panel and to attend the nurses as heretofore. In these circumstances the London Hospital committee have decided not to accept this money for the hospital, but to transfer it to a fund to help London Hospital nurses who from illness may not be able to continue nursing and are without private means. The fund will be utilised, for instance, for the few cases of nurses who may get ill after many years' work, but before they have earned their pension. Incidentally, this latter announcement shows the grave disadvantage of a promised pension which can never be entered upon, or at all, until after the full completion of a fixed period of service.

### SKEGNESS COTTAGE HOSPITAL OPENED.

Last week the Countess of Scarbrough opened the new Cottage Hospital for Skegness and the surrounding district, which has been established as the result of a decision made two years ago. Mr. S. Coetmore Jones, as chairman of the executive committee, explained that the cost of building amounted to £1,450, of which two-thirds has been subscribed. The equipment of the hospital was paid for by a very successful bazaar, which realised £550, or, in other words, enough to leave a balance of £200, which has been used to reduce the building debt to £250. The architect is Mr. F. J. Parkinson, and, which was amusing as well as promising, at the opening ceremony the Chairman stated that they hoped to undertake extensions at an early date. The present building is a nucleus only. The ground floor consists of three single-bedded wards, bathrooms, operating and sterilising rooms, storerooms, and matron's room, kitchen and offices. The matron's, nurses', and servants' bedrooms are on the first floor, and the whole is planned with a view to further building. Nurse Collier has been appointed matron.

### THE OXFORD CONFERENCE.

We are glad to be able to affirm the hope expressed last week that Sir William Osler, Regius Professor of Medicine in the University of Oxford, will deliver the Presidential Address at the Conference of the British Hospitals Association, which opens at Oxford on Thursday, June 26. A Canadian by birth, Sir William, who has just returned from the United States, as his recent visit helps to remind us, knows both Canada and America intimately, for he was once Professor of Medicine at McGill University, Montreal, and later Professor of Clinical Medicine at Pennsylvania, while he has also held a professorship at Maryland University. His "System of Medicine" is already a famous book, and "Counsels and Ideals" are but one instance of the way in which he has never regarded medicine as a science to be isolated by its students, but as part of a living organism, of which institutional life is the first expression, the possibility of progress depending upon the co-relation of doctors, patients, and public. No man is better fitted to strike the right note at a conference of hospital men.

### THIS WEEK'S DRUG MARKET.

Few changes in price have taken place during the week and business continues exceedingly quiet. There is a firm tone in the Quinine market, but before the agreement between the Java cinchona planters and the European quinine makers takes effect several formalities have to be completed. There is a possibility that even at the last moment negotiations may be broken off, but this is not regarded as probable. Some business has been done in Japanese refined camphor, and there is a tendency towards slightly higher prices. The position of cocaine is unchanged. The Cod-liver Oil market is in a stagnant condition, and the price tendency is still downwards. Essence of lemon is again dearer, and menthol is rather lower in price. The value of carbolic acid has a lower tendency.

